# How the Hospital Is Helping in the War

**Published:** 1915-07-17

**Authors:** 


### THE EDITOR'S LETTER-BOX.

### How "The Hospital" is Helping in the War.

*To the Editor of* The Hospital.

Sir,—Some time ago you published in The Hospital the plans of the 5th Northern General Hospital, which was converted into a hospital from the old Leicestershire and Rutland County Asylum.

We have now recently extended this hospital by 530 beds, adopting the open-air wards, and we are sending you by this post a block plan showing the buildings erected up to date; also a detailed plan and section of the new wards. You will see from these that we have introduced some new features which may be of interest to the readers of The Hospital.

The wards appear to answer their purposes quite admirably, and both the staff and the patients are highly delighted with them. The short description gives some particulars; four photographs, also enclosed, explain themselves, and a copy of the Leicester *Daily Post* gives an account of a meeting at which the Mayor of Leicester handed over to the men a bonus earned by the contractors.

The medical staff, nurses, and ourselves are deeply grateful to you for the leader you inserted in your paper in favour of extending the base hospital in preference to adapting a number of schools and other buildings in the town. It was no doubt largely due to this that the authorities were able to persuade the War Office to accept our proposals.

Everybody who is connected with the working of this hospital now fully realises the enormous advantage that has accrued through this extension over that which would have occurred if the separate school buildings had been adopted.—Yours faithfully,

Everard, Son and Pick,

Architects.

6 Millstone Lane, Leicester,
July 9, 1915.

